# Supplementary material for: Evaluation of the Effects of Instant Cascara Beverage on the Brain-Gut Axis of Healthy Male and Female Rats
Source: Nutrients. 2023 Dec 25;16(1):65. doi: 10.3390/nu16010065 (PMC10780800; doi:10.3390/nu16010065)
Supplement: Supplementary file 1 [file nutrients-16-00065-s001.zip › nutrients-2781196-supplementary.pdf]

Supplementary material

Evaluation of the Effects of Instant Cascara Beverage on the Brain-Gut Axis of Healthy Male and Female Rats

Gastrointestinal Motility (Radiographic Study)

Semiquantitative Analysis of Gastrointestinal Motility

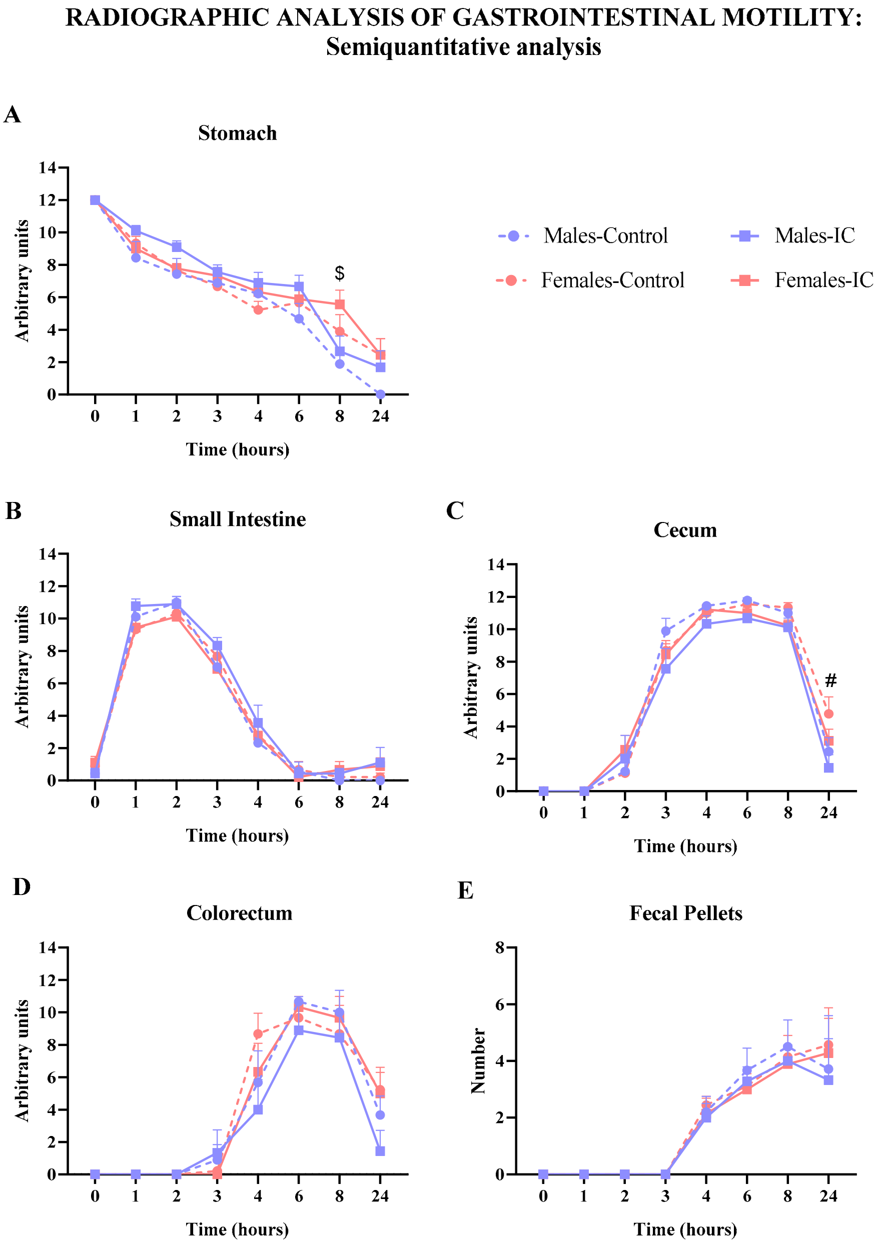

Figure S1. Effect of INSTANT CASCARA (IC) beverage on gastrointestinal transit of male and female rats evaluated radiographically 24 hours after IC administration. X-rays were performed 0, 1, 2, 3, 4, 6, 8, and 24 hours after barium administration. Each gastrointestinal organ receives a numerical value according to the criteria described by Cabezos *et al.* (2008) [29] and the values obtained in the semiquantitative analysis for the stomach (A), small intestine (B), cecum (C) and colorectum (D) are depicted, as well as the number of fecal pellets within the colorectum (E) at each time point. Representative X-rays obtained from males (blue frame, upper panel) and females (red frame, lower

panel) are shown in (F). This acute study of gastrointestinal motility was evaluated after 24 hours of administration of IC in four experimental groups, distributed according to sex and the administered beverage (IC or water): Males-Control, Males-IC, Females-Control and Females-IC. Data represent mean ± SEM (standard error of the mean). n = 12 animals per group. Sex-dependent statistically significant changes: # $p<0.05$ [Females-Control vs Males-Control]; $ $p<0.05$ [Females-IC vs Males-IC]. Two-way ANOVA followed by Bonferroni's post-hoc test.

Morphometric and Densitometric Analysis of Gastrointestinal Organs

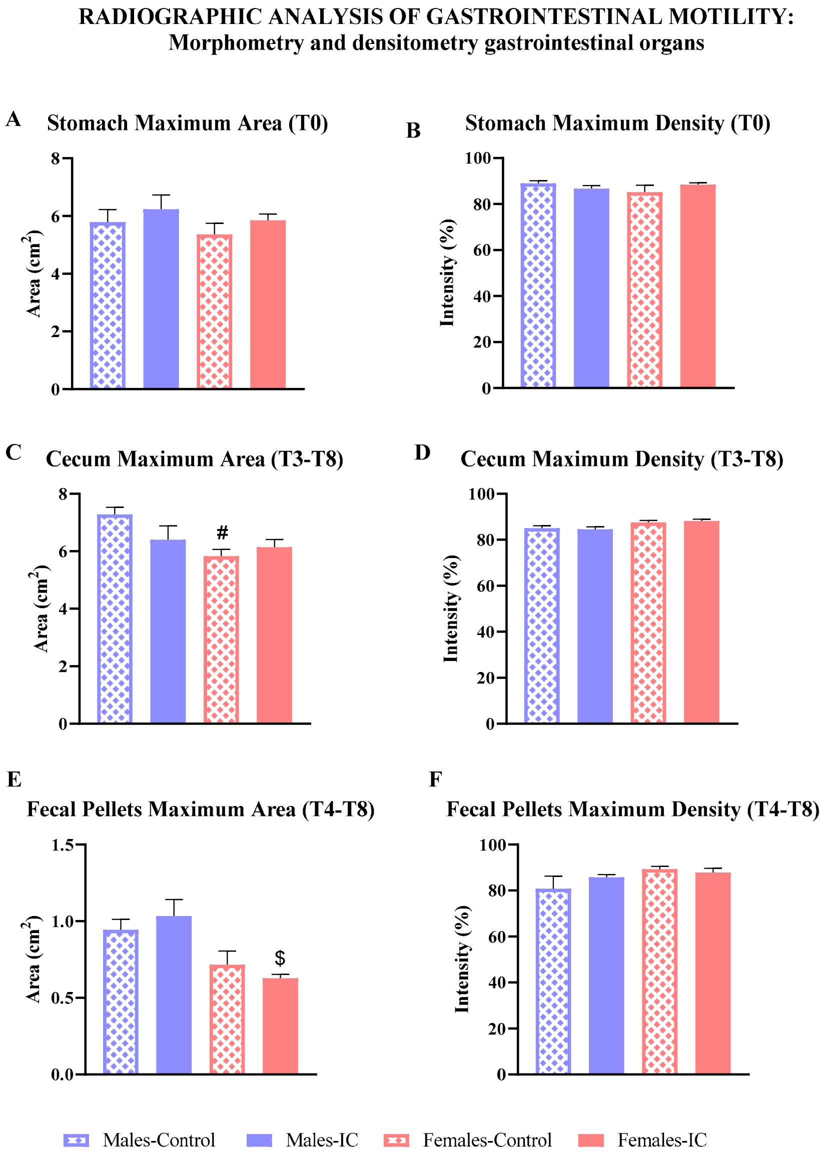

Figure S2. Effect of INSTANT CASCARA (IC) beverage on the morphometric and densitometric analysis of gastrointestinal organs 24 hours after IC administration. Area (A, C, E) and density (B, D, F) of stomach (A, B), cecum (C, D) and fecal pellets (E, F) were measured with IMAGE J, from the X-rays obtained at 0, 1, 2, 3, 4, 6, 8 and 24 hours after barium administration. This study of gastrointestinal motility was evaluated after 24 hours of administration of IC (acute) in four experimental groups, distributed according to sex and the administered beverage (IC or water): Males-control, Males-IC, Females-control and Females-IC. The maximum values for area and barium density were obtained at T0 for the stomach and after averaging the values obtained at T3-T8 for the cecum and at T4-T8 for the fecal pellets. Data represent mean ± SEM (standard error of the mean). n = 12 animals per group. Sex-dependent statistically significant changes: # $p<0.05$ [Females-control vs Males-control]; $ $p<0.05$ [Females-IC vs Males-IC]. One-way ANOVA followed by Bonferroni's post-hoc test.

Analyses of the Feces Collected during the X-ray Session

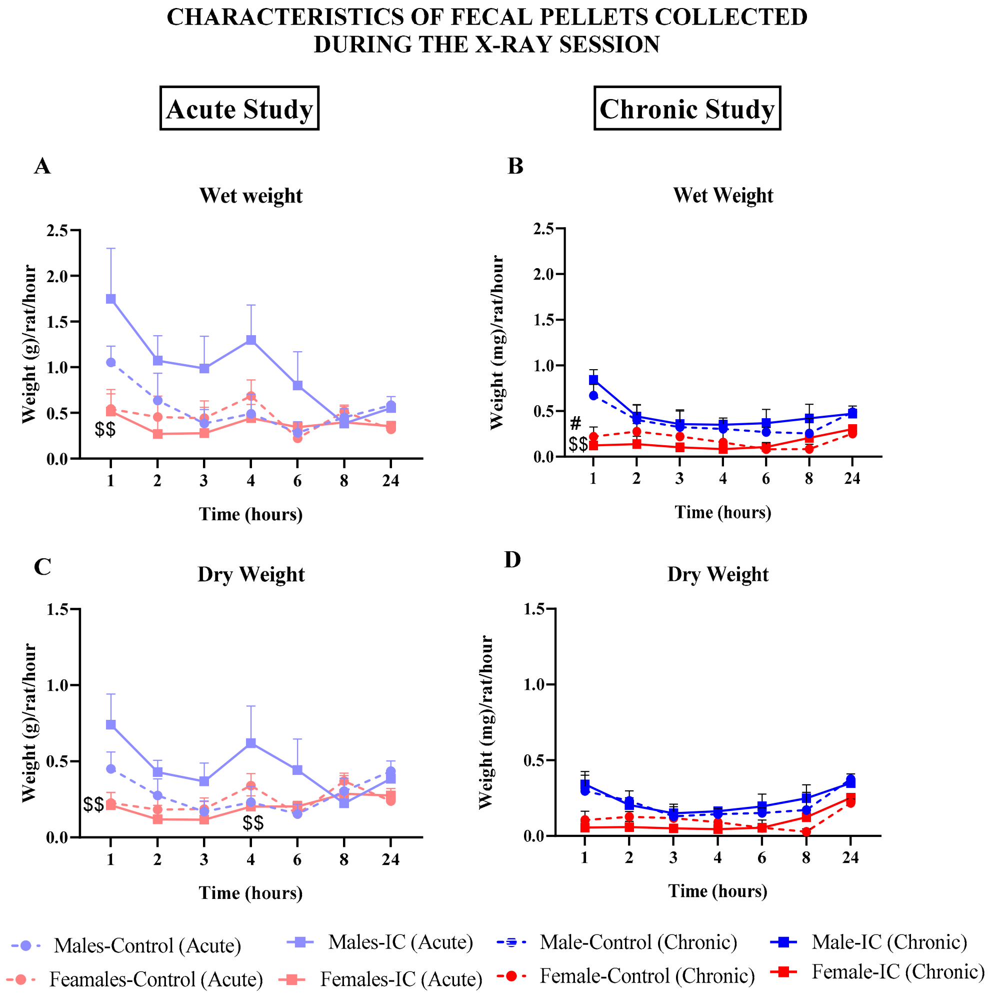

Figure S3. Effect of INSTANT CASCARA (IC) beverage on the feces of male and female rats. Feces were collected from the cages throughout the X-ray session. Once the feces were collected, they were weighed and then placed in an oven at 70ºC for 24-48 h. Wet weight of fecal pellets (A,B) and dry weight of fecal pellets (C,D) are represented. Fecal pellets were collected after 1 day of IC beverage exposure (Acute study, A,C) and in the third week of administration of the IC beverage (Chronic study, B,D). There were four experimental groups, distributed according to sex and the beverage administered (IC or water): Males-Control, Males -IC, Females-Control and Females-IC. Data represent mean ± SEM (standard error of the mean). n = 12 animals per group. Sex-dependent statistically significant changes: # $p<0.05$ [Females-Control vs Males-Control]; $$ $p<0.01$ [Females-IC vs Males-IC]. Two-way ANOVA followed by Bonferroni's post-hoc test.

Macroscopic Analysis

As shown in table S1, at sacrifice females presented statistically significant lower body weight values, regardless of the exposure to water or IC beverage.

The weight values of epididymal/periovarian and retroperitoneal fat, normalized to body weight, did not show any statistically significant differences associated with gender or exposure to IC beverage.

To analyze the weights of the gastrointestinal organs they were also normalized to the weight of the animal. The normalized weight of the organs at sacrifice tended to be higher in control females when compared to control males, although these differences were not statistically significant, except for the colon with feces, in which this difference

was significantly increased in females. In the case of the milking content, it was similar in both sexes.

Regarding the sizes of the gastrointestinal organs, compared to the control males, females presented smaller areas (stomach, cecum) and lengths (small intestine, colon), with the differences in the cecum and length of the colon with feces being statistically significant.

Exposure to IC beverage tended to increase the normalized weight and the size of the organs in both sexes, but this increase was significant only for the cecum area and the length of the colon with feces in males.

Table S1. Effect of INSTANT CASCARA (IC) beverage on the macroscopic characteristics of the gastrointestinal organs and epididymal/periovarian and retroperitoneal fat.

			**Males-Control**	**Males-IC**	**Females-Control**	**Females-IC**
Weight of the animal at sacrifice (g)			316±8	329±7	226±8 (####)	228±6 ($$$$)
Fat (expressed as % of the animal's weight)	EPID/OVA		1.64±0.18	1.56±0.18	1.61±0.14	1.61±0.08
	RETRO		1.64±0.15	1.55±0.13	1.72±0.14	1.68±0.10
Organ weight at sacrifice (expressed as % of the animal's weight)	Stomach		1.43±0.15	1.68±0.16	1.45±0.15	1.70±0.14
	Small intestine	Full	3.08±0.18	3.49±0.16	3.29±0.12	3.35±0.08
		Milking	0.87±0.05	0.98±0.09	0.85±0.08	0.89±0.07
		Empty	2.24±0.06	2.35±0.11	2.35±0.06	2.39±0.04
	Cecum		1.97±0.11	2.14±0.47	2.09±0.12	2.14±0.07
	Colon	With feces	0.95±0.08	1.15±0.14	1.36±0.12 (#)	1.36±0.09
		Without feces	0.62±0.06	0.60±0.08	0.66±0.07	0.69±0.02
Area (cm^2) or length (cm) of organs at sacrifice	Stomach	Area	4.92±0.34	5.63±0.38	3.96±0.25	4.42±0.27 ($)
	Small intestine	Length	60.04±1.99	63.76±1.62	56.17±1.62	59.42±1.87
	Cecum	Area	7.35±0.18	8.57±0.50 (*)	5.85±0.22 (##)	6.14±0.27 ($$$$)
	Colon	Length with feces	12.17±0.47	13.87±0.40 (*)	11.50±0.44 (##)	11.92±0.40
		Length without feces	14.50±0.58	14.54±0.41	12.88±0.56	12.92±0.46

After weighing the animals, they were sacrificed and the gastrointestinal organs were removed *en bloc*, a photograph was taken of the gastrointestinal organs placed on graph paper for the later analysis of the areas/lengths of the different organs with IMAGE J. Once the photograph was taken, the organs were weighed separately on a precision scale and their weights were recorded. In the case of the colon and small intestine, they were weighed with and without their content. The milking corresponds to the content of the small intestine. In addition, epididymal/periovarian fat and retroperitoneal fat were weighed. Weights of fat and gastrointestinal organs are presented as the % of body weight. The parameters were evaluated at sacrifice, at the end of the third week of administration of the IC drink in four experimental groups, distributed according to sex and the administered beverage (IC or water): Males-Control, Males-IC, Females-Control and Females-IC. Values represent means ± SEM (standard error of the mean). n = 12 animals per group. Sex-dependent statistically significant changes: # $p<0.05$, ## $p<0.01$, #### $p<0.0001$ [Females-Control vs Males-Control]; $ $p<0.05$, $$$$ $p<0.0001$ [Females-IC vs Males-IC]. Beverage-dependent statistically significant changes: * $p<0.05$ [Males-IC vs Males-Control]. Statistical analysis: One-way ANOVA followed by Bonferroni post-hoc test.

Vaginal Cytology Smear in Female Rats

Table S2. Distribution of female animals (%) according to their estrous cycle in the different studies performed.

			Proestrus (%)	Estrus (%)	Metestrus (%)	Diestrus (%)
**Behavioral assays**	Cohort 1	Control	17	58	0	25
		IC	27	46	0	27
	Cohort 2	Control	40	40	0	20
		IC	50	33	0	17
**Gastrointestinal studies**	Cohort 1	Control	17	83	0	0
		IC	0	100	0	0
	Cohort 2 (+)	Control	33	17	42	8
		IC	17	58	0	25
**Sacrifice**	Cohort 1	Control	55	27	18	0
		IC	27	18	9	46

Two experimental groups, distributed according to the administered beverage (IC or water) were used: Females-Control and Females-IC. Two cohorts were studied, in which different studies were performed. In cohort 1, the behavioral assays were splash test and hole-board test and the radiographic study of gastrointestinal motility was performed just before these studies, at the beginning of week 3 of IC exposure; the organs of these rats were also studied at sacrifice, at the end of the study. In cohort 2, the behavioral assay was the plus-maze test, performed during week 3, and the gastrointestinal motility study was performed just one day after starting the exposure to IC. Data represent mean ± SEM. Beverage-dependent statistically significant changes: + $p<0.05$ [Females-IC vs Females-Control]. Chi square test.
